# The impact of digital health literacy on marriage-and-childbearing anxiety among Chinese youth of reproductive age

**DOI:** 10.3389/fpsyg.2025.1676542

**Published:** 2025-11-27

**Authors:** Hao Li, Jiao Li, Zihan Yang

**Affiliations:** 1 School of Business, Xinyang Normal University, Xinyang, China; 2 Research Institute of the Economic and Social Development in the Dabie Mountains, Xinyang, China

**Keywords:** digital health literacy, marriage-and-childbearing anxiety, information cognition, risk perception, reproductive-age youth

## Abstract

**Introduction:**

In the digital era, information on marriage and childbearing is predominantly accessed online, yet the coexistence of information overload and misinformation may amplify anxiety among reproductive-aged young adults. This study investigates the impact and underlying mechanisms of digital health literacy (DHL) on marriage-and-childbearing anxiety (MCA), aiming to provide empirical evidence for alleviating this growing concern.

**Methods:**

An empirical analysis was conducted using survey data from 748 reproductive-aged young adults. Multiple econometric methods were employed to ensure robustness: ordinary least squares (OLS) regression and ordered Probit models for baseline estimates, instrumental variable two-stage least squares (2SLS) to address endogeneity, and formal mediation tests to examine indirect effects.

**Results:**

First, DHL exhibits a significant negative effect on MCA, which remains stable after robustness checks and endogeneity correction. Second, information cognition, risk perception, and social support serve as three key mediating pathways linking DHL to MCA, with risk perception playing the most prominent role. Third, the protective effect of DHL against MCA shows significant heterogeneity across gender, household registration type, income level, urban classification tiers, and different dimensions of DHL.

**Discussion:**

These findings elucidate the mechanisms through which DHL influences MCA and provide targeted theoretical and empirical support for developing evidence-based interventions to reduce marriage-and-childbearing anxiety among young adults in the digital era.

## 1 Introduction

Rapid advances in information technology have ushered society into a digital epoch defined by ubiquitous connectivity, exponential data growth and instantaneous dissemination. Social media, online medical platforms and health-management applications have fundamentally reshaped how individuals seek information, interact and organize daily life. Within this context, digital health literacy (DHL) denotes the set of competencies required to locate, evaluate and apply health-related information in digital environments (Van Der Vaart and Drossaert, 2017). High DHL enhances autonomous health management, ensures timely access to reliable information and supports evidence-based decision-making, thereby improving health outcomes (Li and Yang, 2024).

Concurrently, marriage and childbearing anxiety (MCA) among reproductive-age youth has become increasingly salient. Despite familial and societal expectations, many young adults exhibit avoidance, anxiety and resistance toward marriage and parenthood. National Bureau of Statistics data show that China’s total fertility rate has long remained below the replacement level of 2.1; in 2023, new-borns reached a historical low, while the average age at first marriage rose from 24.89 years (2010) to 28.67 years (Ren Zeping Team, 2024). Structural constraints such as million-yuan parenting costs and work–family conflict under “996” schedules interact with amplified childhood-trauma narratives circulating in digital media. Youth express substantial concerns over economic responsibilities in marriage, partner relations, household-role allocation, post-childbearing costs, career disruption and lifestyle degradation (Fiskin and Sari, 2021). This psychological stance reshapes individual life planning and family formation, with profound implications for demographic structure, family paradigms and sustainable social development (He et al., 2024).

It is noteworthy that the “digital environment” is neither a monolithic nor homogeneous space. Significant differences exist in the platform architectures and communication logics through which young people access marriage- and childbearing-related information (Tao and Yang, 2025). For instance, algorithm-driven short-video platforms (e.g., Douyin, Kuaishou) tend to push fragmented content with high emotional value; search-based platforms (e.g., Baidu) prioritize information retrieval efficiency; and community-based platforms such as Douban and Zhihu focus on in-depth discussions and circle-based interactions. The characteristics of information overload and misinformation also vary across different platforms. Such differences not only lead to uneven distribution in the presentation formats, dissemination speeds, and credibility of marriage- and childbearing-related information but also collectively shape young people’s information exposure patterns through algorithmic recommendation mechanisms and platform functional designs (Nettasinghe et al., 2024). DHL is precisely the key competence that enables young people to navigate these complex information ecosystems across platforms, and its role thus gains more specific contextual support.

In existing research on alleviating low fertility, attention has mostly focused on macro-level measures such as fiscal incentives and childcare service provision, while the psychological mechanisms underlying youth marriage-and-childbearing anxiety (MCA) in the digital era and corresponding digital solutions have been generally overlooked, which constitutes a key gap in current studies (Berlinski et al., 2024). To address this gap, the present study takes DHL as its core entry point and, based on the theoretical framework of DHL, systematically explores the relationship, operational pathways, and heterogeneous effects across demographic groups between DHL and MCA among Chinese youth of reproductive age.

First, the study delineates the prevailing levels of DHL and MCA within this cohort, further unpacks the psychological, social, and economic logics underlying the correlation between the two, and makes two key contributions to extant literature. Theoretically, whereas prior DHL research has focused on conceptual delineation and general health behaviours, this study is the first to apply the concept to MCA among young adults: it extends the application scenario of DHL from traditional chronic disease management to the field of demographic behavior, expands the analytical boundary of DHL, enriches the connotation of health information behavior theory, and offers a novel theoretical lens on youth psychology in digital contexts. Methodologically, the study constructs a multidimensional indicator system for DHL and MCA, integrates a specially designed questionnaire survey, and combines survey data with rigorous econometric methods—including ordinary least squares (OLS), ordered Probit, instrumental variable (IV) estimation, and mediating effect tests. Through these approaches, the study not only clarifies the causal impact of DHL on young people’s phobic attitudes toward marriage and childbearing and identifies the corresponding relationships between each dimension of DHL and distinct anxiety determinants, but also provides robust causal evidence on the DHL–MCA nexus. Policy-wise, the findings yield actionable insights for precision DHL education and psychological counseling: the study proposes targeted digital empowerment strategies that offer a new practical pathway for optimizing marriage-and-childbearing support policies, alleviating youth MCA, and informing policymakers and NGOs in designing marriage- and family-support services, thus thereby contributing to both theoretical advancement and policy design.

## 2 Conceptual framework and theoretical basis

### 2.1 Digital health literacy: definition and dimensions

DHL is defined as the ability to seek, find, understand, appraise and apply health information from electronic sources to address specific health problem (Norman and Skinner, 2006). The construct embeds the interactive use of digital devices, algorithmic recommendations and heterogeneous data within the traditional health-literacy framework, emphasizing critical and adaptive decision-making within complex information ecosystems.

DHL encompasses four interrelated core dimensions that collectively support individuals in navigating the digital health information environment. The first dimension, information acquisition, refers to the ability to efficiently locate relevant health information through search engines, health apps, and online platforms, while also conducting preliminary assessments of the credibility of information sources (Van Der Vaart and Drossaert, 2017). Building on this foundational ability, the second dimension, information evaluation, involves critically appraising the quality of acquired information based on criteria such as accuracy, scientific rigor, timeliness, and source authority (Estrela et al., 2023). The third dimension, information application, focuses on translating validated and reliable information into concrete health-related decisions or behaviors, which includes modifying lifestyles, enhancing doctor-patient communication, and improving adherence to treatment plans (Yang et al., 2022). Beyond information-focused capacities, DHL also incorporates a risk prevention dimension, which entails identifying and mitigating potential risks in the digital health sphere, such as privacy breaches, exposure to misinformation, and vulnerability to commercial fraud (Van Kessel et al., 2022).

Thus, DHL is a highly contextualized competence. It not only enhances individuals’ health autonomy but also provides a novel theoretical perspective for marriage and childbearing decision-making. Its core value and effectiveness are closely dependent on users’ understanding and adaptation to the algorithmic logics, information presentation formats, and community norms of specific platforms (e.g., social media, health applications, search engines) (Yuliana et al., 2025). DHL enables individuals to accurately adapt to the information ecosystems of different platforms: it supports efficient screening of valid information from algorithm-recommended fragmented content, establishment of rational judgments amid diverse viewpoints in community discussions, and rapid localization of authoritative sources among the massive results of search engines. Ultimately, this facilitates the scientific control and rational utilization of marriage- and childbearing-related information.

### 2.2 Anxiety about marriage and childbearing: concepts, characteristics and manifestations

MCA denotes a persistent state of fear, worry and avoidance experienced by reproductive-age individuals who anticipate that marriage and childbearing will impose unbearable responsibilities, costs and uncertainties (Sönmez and Aktaş, 2023). This psychological state constitutes a pronounced barrier that distorts romantic cognitions and disrupts family-formation plans. Its genesis lies in excessive apprehension toward the manifold obligations, pressures and ambiguities associated with marital and parental roles (Matinmanesh and Sharifi, 2021).

The clinical presentation of MCA can be taxonomised into four interrelated domains that collectively shape individuals’ attitudes and behaviors toward marriage and childbearing. The first domain centers on responsibility avoidance, which involves hypersensitivity to the economic obligations associated with home ownership, child-rearing, and eldercare, as well as catastrophic expectations regarding potential marital conflict and intergenerational tension (Chang, 2024). The second domain is cost overestimation, referring to the magnification of direct economic costs such as those related to marriage, maternity, education, and housing, alongside opportunity costs including career interruption and loss of leisure, while the emotional rewards of marriage and childbearing are discounted (Chung and Lee, 2022). The third domain encompasses behavioral avoidance, manifesting as topic diversion, emotional withdrawal, or even relationship termination when partnerships approach the stage of marriage or reproduction, along with active information blocking in digital contexts (Xiong et al., 2022). The fourth domain involves negative quality-of-life forecasting, where individuals equate post-marital and post-natal life with “freedom deprivation” and “dilution of happiness,” thereby reinforcing a pessimistic orientation toward the future (Othman, 2024). These four manifestations mutually reinforce one another through a cognitive–emotional–behavioral feedback loop, ultimately dampening fertility intentions.

### 2.3 The transmission path of MCA influenced by DHL

#### 2.3.1 Information cognition pathway

At the cognitive level, DHL attenuates MCA by reconfiguring how young adults process marriage- and childbearing-related information (Estrela et al., 2023). High-DHL individuals integrate authoritative medical websites, evidence-based applications and online platforms to obtain balanced knowledge on married life, antenatal care, reproductive technologies and parenting strategies (Li and Yang, 2024). Their critical-appraisal skills enable them to filter rumors and sensational narratives, reducing catastrophising appraisals. Conversely, individuals with low DHL, constrained by limited information sources and weak evaluative capacity, are more likely to internalize one-sided or hyperbolic negative content, which fosters risk overestimation and avoidance tendencies (He et al., 2024). Furthermore, algorithmic recommendation mechanisms amplify the one-sidedness of information through the “information echo chamber” effect, tending to push negative narratives or extreme cases that align with users’ interests (Zhou, 2025). This makes individuals with low DHL more likely to be dominated by algorithms’ emotional recommendations, trapped in a unidimensional information environment, and to internalize one-sided or exaggerated negative content, thereby reinforcing cognitive biases. In contrast, individuals with high DHL can sensitively identify information echo chambers or emotional polarization potentially caused by algorithmic recommendations, proactively break free from algorithmic constraints, and integrate diverse resources such as authoritative medical websites and evidence-based applications across different platforms (Yuliana et al., 2025). Through cross-validation, they overcome the limitations of “echo chambers,” ultimately acquiring balanced knowledge about marital life and parenting strategies and forming rational and objective cognitive judgments.

#### 2.3.2 Risk perception pathway

DHL reduces MCA by enhancing the precision of risk perception and the perceived controllability of marriage- and childbearing-related challenges (Taba et al., 2022). High-DHL respondents systematically identify physiological, psychological and economic dimensions of the transition, then assign realistic probabilities and controllability ratings, reframing the transition as a “manageable challenge” (Li and Yang, 2024). Proactive behaviors such as preconception planning and evidence-based budgeting bolster coping self-efficacy and reduce anxiety. In contrast, low-DHL individuals disproportionately amplify negative cues and perceive challenges as uncontrollable, fostering helplessness and intensifying anxiety (He et al., 2024). The engagement maximization logic of algorithmic recommendations tends to amplify marriage and childbearing-related risk topics, leading individuals with low DHL to develop an overperception of potential challenges (Yuliana et al., 2025). In contrast, individuals with high DHL can sensitively identify such biased recommendations by algorithms and the phenomenon of algorithm-induced risk signal amplification. Through scientific evaluation, they reduce the “perceived risks” exaggerated by algorithms to “actual risks.” Furthermore, they systematically identify multi-dimensional challenges such as physiological, psychological, and economic aspects during the transition to marriage and childbearing, assign objective probability assessments to these challenges, and formulate targeted response strategies (Berlinski et al., 2024). This process significantly enhances their sense of controllability over the relevant risks.

#### 2.3.3 Social support pathway

DHL mitigates MCA by expanding both the quantity and quality of social support (Estrela et al., 2023). High-DHL young adults leverage digital communities and professional platforms to obtain emotional support, information and expert guidance, constructing “weak-tie, strong-support” networks that buffer stress (Li and Yang, 2024). Participation in online parenting forums and tele-counseling provides practical strategies and enhances self-efficacy through social modeling. In contrast, low-DHL individuals face platform-navigation barriers and limited help-seeking knowledge, confronting pressures in isolation and sustaining elevated anxiety (Sönmez and Aktaş, 2023). DHL mitigates MCA by expanding both the quantity and quality of social support; however, the supportive function of online communities exhibits a distinct dual nature (Das, 2023). On the one hand, individuals with high DHL can accurately connect with professional platforms and positive mutual-aid communities, obtain practical advice and emotional resonance, and construct “weak-tie, strong-support” networks. On the other hand, online communities may also serve as sources of social comparison, judgment, and even misinformation (Sönmez and Aktaş, 2023). Issues such as the culture of comparison and the spread of extreme viewpoints existing in some communities may further intensify the pressure from social comparison. DHL, however, equips individuals with the key ability to screen high-quality communities, block negative information, and avoid toxic interactions, allowing them to effectively mitigate the potential risks of online support and maximize the benefits of positive support (Van Kessel et al., 2022). This also explains why the effect of digital health literacy (DHL) on alleviating perceived social pressure is relatively limited, as the dual attributes of online communities restrict its role in offsetting structural social pressure to a certain extent (Das, 2023).

**Figure 1:**
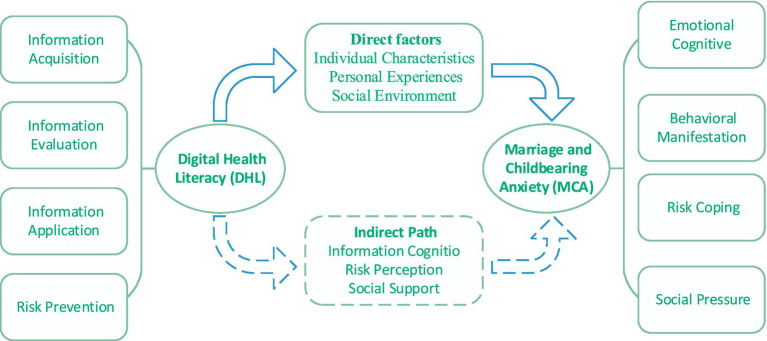
DHL affects MCA mechanism pathway.

The mediating pathways through which DHL influences MCA are depicted in Figure 1.

## 3 Empirical analysis

### 3.1 Data collection

In 2024, we conducted an online survey targeting unmarried Chinese adults aged 18 to 35. The study adopted a multi-platform online sampling strategy to ensure geographic and demographic coverage. Questionnaires were distributed via the Wenjuanxing platform (a professional survey tool widely used in Chinese academic research) and disseminated through channels including WeChat official accounts, Weibo topic sections, and Douban lifestyle groups. This approach aimed to maximize geographic and demographic coverage and to include young people with diverse interests and information exposure habits to the greatest extent possible. The use of multiple platforms in sample recruitment helped reduce sampling bias caused by the characteristics of users from a single platform. However, differences in user profiles across various platforms may still have potential impacts on the results.

Of the 1,000 questionnaires distributed, 748 were valid, corresponding to an effective response rate of 74.8%; IP-based geolocation indicated that the sample covers multiple provinces in eastern, central, and western China, including first- to fourth-tier cities and rural areas, with the following demographic characteristics: age ranging from 18 to 35 years (24.6% aged 18–22, 31.8% aged 23–28, and 43.6% aged 29–35), a balanced gender distribution (48.1% male and 51.9% female), and educational attainment distributed across high school or below (7.7%), junior college (12.7%), bachelor’s degree (59.4%), and master’s degree or higher (20.2%); overall, the demographic structure (encompassing age, gender, and education) is basically consistent with the data on the 18–35-year-old youth group released by the National Bureau of Statistics, indicating good sample representativeness.

### 3.2 Current status of young adults’ DHL

The DHL scale was adapted from the Health Information Literacy Self-Rating Scale developed by Norman and Skinner (2006), Van Der Vaart and Drossaert (2017), and Estrela et al. (2023). This original scale is culturally applicable to Chinese populations, as it was developed based on the health information literacy framework proposed by the U. S. National Library of Medicine and validated among over 2,000 Chinese adults (Yang et al., 2022). To align with the study’s focus on “digital contexts” and “MCA scenarios,” we retained 12 core items and revised the wording to reflect MCA-related information needs, without altering the original conceptual connotations. The adapted scale comprises three dimensions: Information Acquisition, Information Evaluation, and Information Application. The original scale has been widely used in Chinese health behavior research and demonstrated robust psychometric properties, which ensures the validity of the adapted version (Yang et al., 2022). Specific indicators appear in Table 1.

**Table 1:** Comprehensive indicator system for DHL measurement.

Primary indicator	Secondary indicator	Tertiary indicator
Information acquisition capacity	Channel siversity	Ability to identify authoritative platforms
		Professional database search competence
	Query precision	Keyword optimization proficiency
		Multi modal information acquisition capability
Information evaluation capacity	Credibility assessment	Authoritative analysis of information sources
		Business promotion information recognition
	Scientific analysis	Ability to distinguish research methods
		Data interpretation ability
Information application capacity	Health decision implementation	Individualized health plan development
		Medical resource coordination capability
	Health behavior translation	Digital tool assisted management capability
		Health data integration capability
Digital health risk protective awareness	Proactive privacy protection	Ability to identify sensitive information
		Data authorization management capability
	Risk avoidance strategies	Identification of health fraud information
		Psychological health protection mechanism

Using a five-point Likert scale (1 = completely unable; 5 = completely proficient) and 16 items, we standardized scores and derived weights via the entropy method. The composite DHL mean is 0.8654, indicating above-average literacy. Sub-domain means (out of 20) are: acquisition 13.4, evaluation 12.8, application 13.2 and risk prevention 8.9. Subgroup analyses reveal that respondents aged 18–22 outperform older cohorts in technical operations but score lower on evaluation, underscoring the moderating role of life experience. Educational attainment exhibits a pronounced gradient, with bachelor’s or above outperforming high-school or below across all dimensions.

### 3.3 Construction of the MCA index

The MCA scale used in this study is a comprehensive measurement framework that integrates two contextually adapted and validated Chinese scales as well as two complementary assessment tools embedded in the questionnaire. It aims to systematically measure the MCA level of young people while comprehensively capturing anxiety related to marriage and childbearing. Specifically, the scale includes two core subdimensions: the 6-item Marital Anxiety Subdimension was adapted from the Marital Pressure Scale for Chinese Urban Residents developed by Spanier (1979), an instrument validated among Chinese young people that focuses on culturally specific marital stressors such as economic pressure and intergenerational conflict; we incorporated digital-era characteristics and added a separate self-assessment item (“To what extent do you feel fearful about marriage/childbearing?”) scored on a Likert scale (1 = “Not at all,” 5 = “Extremely obvious”). The 6-item Fertility Anxiety Subdimension was adapted from the Fertility Anxiety Questionnaire for Chinese Reproductive-Age Populations by He et al. (2025), a recent scale targeting fertility-related worries among Chinese young people that covers cost, health, and social pressure; we adjusted its wording to link it with digital information exposure, drawing on multidimensional anxiety items derived from popular topics on major social media platforms, dating forums, and professional survey portals. The aforementioned items were refined to form the MCA index presented in Table 2, which is scored on a 5-point Likert scale with weights derived from entropy, higher scores indicate higher levels of anxiety.

**Table 2:** Comprehensive indicator system for measuring MCA.

Primary indicator	Secondary indicator	Tertiary indicator
Emotional cognitive dimension	Fear of intimacy	Avoidance of emotional connection
		Distrust in partner
		Resistance to long-term commitment
	Self-worth concerns	Anxiety about marital/parental role competence
		Appearance/ability depreciation anxiety
		Low parenting self-efficacy
	Freedom loss Anxiety	Fear of personal space compression
		Concerns about independent decision-making
		Resistance to lifestyle changes
Behavioral manifestation dimension	Marital/parenting topic avoidance	Refusal to discuss wedding/childbearing plans
		Postponement of wedding dates
		Avoidance of intimate contact
	Physiological stress response	Pre-marital insomnia/nightmares
		Somatic symptoms (headache, palpitations)
		Emotional outburst frequency
	Over-preparation behaviors	Excessive saving (economic risk prevention)
		Compulsive prenuptial agreement drafting
		Frequent partner switching due to commitment anxiety
Social pressure dimension	Parental family influence	Projection of parental marital patterns (conflict/divorce)
		Intensity of family pressure to marry
		Degree of financial dependence
	Sociocultural internalization	Endorsement of “marriage must lead to childbirth” concept
		Pressure from stereotypical gender roles
		Social comparison anxiety
	Perceived economic risks	Wedding cost pressure
		Estimated childrearing economic burden
		Career interruption anxiety
Risk coping dimension	Responsibility avoidance	Presumption of refusing household chores
		Outsourcing parenting responsibility
		Conflict resolution avoidance strategies
	Negative outcome amplification	Overestimation of divorce probability
		Catastrophizing childbirth outcomes
		Hyperfocus on failed marriage cases
	Inadequate support systems	Reluctance to seek help
		Prevalence of marriage-fear in social circles
		Utilization rate of professional psychological resources

The composite MCA mean is 0.7851, indicating elevated anxiety. By dimension: emotional-cognitive 23.4, behavioral 18.1, social-pressure 32.8, risk-coping 24.3. Anxiety increases with age and is significantly higher among bachelor’s or above, arguably reflecting the gap between idealized expectations and perceived realities.

### 3.4 Reliability and validity test of DHL and MCA scales

To ensure the scientificity and credibility of the constructed composite indices (DHL and MCA), we conducted comprehensive quality tests on the scales, including reliability, validity, and common method bias (CMB) tests. Reliability was evaluated using Cronbach’s *α* coefficient and composite reliability (CR) to verify the internal consistency of the scale items: the DHL scale has a Cronbach’s α coefficient of 0.876, with CR ranging from 0.823 to 0.901 and average variance extracted (AVE) ranging from 0.512 to 0.634; the MCA scale has a Cronbach’s α coefficient of 0.902, with CR ranging from 0.856 to 0.924 and AVE ranging from 0.537 to 0.658, all meeting the reliability and validity test standards.

Validity was assessed from two dimensions: convergent validity (measured by AVE) and discriminant validity (verified by comparing the square root of AVE with inter-dimensional correlation coefficients); exploratory factor analysis shows that all item factor loadings are greater than 0.6, and the discriminant validity of each dimension is satisfied (the square root of AVE is greater than the inter-dimensional correlation coefficient).

Given that all measurement items (including DHL, MCA, and control variables) were collected via self-reported questionnaires, we further adopted Harman’s single-factor test to address potential CMB. The unrotated exploratory factor analysis (EFA) results show that the variance explained by the first common factor is 32.7%, which is lower than the critical threshold of 40%, indicating no severe common method bias in this study. The detailed reliability, validity, and CMB test results are shown in Appendix Tables 1, 2.

### 3.5 Model specification

To examine the relationship between DHL and MCA among surveyed young adults, we estimate the following baseline model 1 (Xiong et al., 2022; Othman, 2024):


(1) $$\mathrm{Mc}a_{\mathrm{ij}}=\alpha +\beta_{1}\mathrm{Dh}l_{\mathrm{ij}}+\sum \theta X_{\mathrm{ij}}+\lambda_{j}+\varepsilon_{\mathrm{ij}}$$

Where, *Mca* denotes the degree of MCA for respondent *i*. *Dhl* denotes the individual’s level of DHL. X is a vector of covariates capturing personal characteristics, life experiences, social environments and economic constraints. λ represents city fixed effects to account for unobserved regional heterogeneity. ε represents the stochastic disturbance term in the model.

### 3.6 Control variables

To isolate potentially confounding influences on MCA, we incorporated control variables across four theoretically grounded domains: individual characteristics, personal experiences, social environment, and economic pressure (Khojeh et al., 2024). Specifically, the control variables include age, gender, education level, hukou type, employment status, income level, and city tier. They were measured using standard demographic items consistent with Chinese social science surveys. These items have been widely adopted in population and marriage-related research in China, which ensures the comparability and validity of our measurements (Matinmanesh and Sharifi, 2021).

The control variables mainly cover the following four aspects. First, individual characteristics encompass gender (binary), age measured in completed years, highest educational credential attained, household registration type distinguishing urban from rural hukou, and a self-assessment of physical appearance recorded on an 10-point scale (0–10) with higher scores denoting greater satisfaction. Second, personal experiences are captured through four psychometrically validated instruments that record (a) the respondent’s perception of the quality of the parents’ marriage, (b) the cumulative incidence and subjective severity of previous romantic setbacks, (c) enduring dispositional tendencies toward avoidant attachment, and (d) perfectionist expectations regarding future marriage and childbearing. Third, the social environment is operationalized along two sub-dimensions: (a) social norms, indexed by the frequency of exposure to negative online narratives about marriage (never = 0 to daily = 5), and (b) social support, constructed as a composite measure that aggregates the intensity of family assistance, parents’ acceptance of the respondent’s reproductive decisions, and expressed satisfaction with the marriage- and childbearing-related support provided by family and friends. Finally, economic pressure is proxied by the local housing-price-to-income ratio, respondents’ estimates of expected marriage and child-rearing expenditures, perceived income stability, and the adequacy of social-security coverage (Khojeh et al., 2024). Descriptive statistics for all variables are presented in Table 3.

**Table 3:** Descriptive statistics of key variables.

Variable	Symbol	Measurement	Mean	SD
Marriage and Childbirth anxiety	MCA	Comprehensive indicator system	0.7851	2.0324
Digital health literacy	DHL	Comprehensive indicator system	0.8654	2.6548
Age	AGE	Actual age (years)	27.2115	4.6245
Gender	GEN	Dummy variable	0.5187	0.2498
Appearance anxiety	APA	Scale 1–5 (1 = no anxiety, 5 = extreme anxiety)	3.5984	0.9332
Education level	EDU	Actual years of schooling	12.2358	4.2441
Employment status	EMP	Dummy variable (unemployed = 0, employed = 1)	0.7281	0.4449
Household registration	URB	Dummy variable (rural = 0, non-rural = 1)	0.7954	0.6441
Parental marital quality	PMQ	Scale 1–5 (1 = severe conflict, 5 = highly harmonious)	3.4542	1.1237
Romantic relationship setbacks	RRS	Event count (severe frustrations)	2.3472	1.8561
Avoidant personality tendency	APT	Scale 1–5 (Social Avoidance Index)	2.7851	2.4569
Perfectionism in Marriage	PME	Scale 1–5 (1 = no expectation, 5 = extremely perfectionistic)	4.1565	0.8824
Negative information exposure	NIE	Days per week exposed to negative marital information	3.2541	1.5145
Family support level	FSL	Scale 1–5 (1 = no support, 5 = strong support)	3.7521	1.0254
Parental Open-mindedness	POP	Scale 1–5 (1 = conservative, 5 = highly open)	3.6254	1.1854
Relatives’ marital satisfaction	RFS	Average satisfaction rating of 3–5 close relatives (scale 1–5)	3.3854	0.9654
Income stability	INS	Ordered variable (1 = no income, 5 = state-owned/salary-stable)	2.6554	1.8541
Housing Price-to-Income Ratio	HIR	Local average house price ÷ per capita disposable income	12.4342	3.7821
Expected wedding cost	EWC	Anticipated wedding expenses	28.6211	16.2654
Expected childrearing cost	ECC	Estimated total cost for ages 0–18	185.3231	200.8542
Social insurance	SIN	Dummy variable (1 = covered)	0.6925	0.4616

1. All continuous variables are reported with mean (Mean) and standard deviation (Std. Dev.); categorical/ordered variables are reported with mean (proportional or scaled mean) and standard deviation. 2. The sample covers multiple provinces in eastern, central, and western China, including first- to fourth-tier cities and rural areas; the demographic structure (age, gender, education) is basically consistent with the 18–35-year-old youth group data released by the National Bureau of Statistics, indicating good representativeness. 3. The measurement of composite indices (MCA, DHL) adopts the entropy weight method, with scores standardized to the range [0,1]; higher scores for MCA indicate greater anxiety, and higher scores for DHL indicate higher digital health literacy.

## 4 Empirical results

### 4.1 Baseline estimation

Following model (1), we estimate the effect of DHL on MCA via a stepwise OLS procedure. Column (1) presents the most parsimonious specification, with DHL as the sole regressor; Column (2) augments this baseline by adding city fixed effects to absorb unobserved regional heterogeneity. We then sequentially introduce four blocks of controls: demographics, personal experiences, social environment, and economic pressures, while retaining city fixed effects throughout to account for unobserved urban heterogeneity. Variance-inflation factors remain below conventional thresholds, indicating no harmful multicollinearity. The fully specified estimates are reported in Table 4.

**Table 4:** Effect of DHL on MCA.

Variable	(1)	(2)	(3)	(4)	(5)	(6)
DHL	−0.132^***^	−0.137^***^	−0.142^***^	−0.144^***^	−0.151^***^	−0.148^***^
	0.023	0.024	0.031	0.031	0.035	0.035
	(−5.74)	(−5.71)	(−4.58)	(−4.65)	(−4.31)	(−4.23)
	β = −0.215	*β* = −0.223	*β* = −0.230	*β* = −0.233	β = −0.245	β = −0.238
	[−0.177, −0.087]	[−0.184, −0.090]	[−0.203, −0.081]	[−0.205, −0.083]	[−0.220, −0.082]	[−0.217, −0.079]
AGE			0.035^***^	0.036^***^	0.034^***^	0.033^***^
			(3.50)	(3.60)	(3.40)	(3.67)
GEN			0.354^*^	0.354^*^	0.341^**^	0.340^**^
			(1.85)	(1.85)	(1.97)	(1.96)
APA			0.324^***^	0.331^***^	0.324^***^	0.319^***^
			(2.97)	(2.69)	(2.79)	(3.13)
EDU			−0.028^**^	−0.033^**^	−0.041^**^	−0.039^**^
			(−2.15)	(−2.36)	(−2.28)	(−2.44)
EMP			−0.087^***^	−0.108^***^	−0.112^***^	−0.113^***^
			(−3.48)	(−4.32)	(−4.67)	(−4.71)
URB			0.017^***^	0.017^***^	0.021^***^	0.023^***^
			(4.25)	(4.25)	(3.50)	(3.29)
PMQ				−0.657^***^	−0.665^***^	−0.666^***^
				(−7.64)	(−5.73)	(−5.69)
RRS				2.098^***^	2.098^***^	2.104^***^
				(4.66)	(4.74)	(4.35)
APT				0.698^***^	0.699^***^	0.688^***^
				(5.02)	(5.02)	(4.56)
PME				0.057^***^	0.045^***^	0.043^***^
				(4.07)	(3.75)	(3.58)
NIE					1.026^***^	1.026^***^
					(4.58)	(4.59)
FST					−0.247^***^	−0.224^***^
					(−2.98)	(−3.07)
POP					−0.047^***^	−0.044^***^
					(−4.70)	(−4.40)
RFS					−0.024^***^	−0.030^***^
					(−3.43)	(−3.33)
INS						−0.247^***^
						(−5.26)
HIR						2.074^***^
						(4.36)
EWC						1.098^***^
						(3.54)
ECC						2.031^***^
						(4.24)
SIN						−0.321
						(−0.65)
Cons	−0.176^***^	−0.166^***^	−0.318^***^	−0.323^***^	−0.284^***^	−0.247^***^
	(−7.04)	(−6.92)	(−6.63)	(−5.98)	(−4.58)	(−4.19)
Urban FE	NO	YES	YES	YES	YES	YES

***p < 0.01, **p < 0.05, *p < 0.1. The first row presents the regression coefficients between DHA and MCA. The second row shows the standard errors of the estimated coefficients, and the third row reports the t-values (in parentheses). The fourth row displays the standardized estimated coefficients, while the fifth row provides the 95% confidence intervals for the regression coefficients. Due to space constraints, this paper only reports detailed regression results for the key variables DHA and MCA; detailed results for other variables are available from the authors upon request.

Across all columns, DHL exerts a consistently negative and statistically significant effect on MCA. In the most parsimonious model (Column 1), the coefficient on DHL is significantly negative at the 1% level. Augmenting the specification with city fixed effects (Column 2) leaves the estimate virtually unchanged, confirming that the relationship is not driven by unobserved inter-city heterogeneity. Columns 3–6 sequentially introduce four blocks of controls: demographics, psychosocial experiences, social environment, and economic pressures, yet the coefficient of DHL remains stable in sign and magnitude. These results align with health-empowerment theory: individuals with high DHL achieve a “sense of cognitive mastery” by systematically seeking, appraising and applying information, thereby transforming diffuse “unknown risks” into quantifiable and manageable contingencies (Taba et al., 2022). In practice, high-DHL individuals (a) consult authoritative sources to debunk marriage- and fertility-related misinformation; (b) implement evidence-based marital and parenting plans drawn from official health platforms to minimize decisional uncertainty; and (c) deploy digital tools—such as menstrual-cycle-tracking applications—to optimize health management and alleviate pregnancy-related anxiety (Li and Yang, 2024; Xiong et al., 2022; Othman, 2024).

Turning to Column 6, age carries a positive coefficient, indicating that the narrowing reproductive window intensifies anxiety as individuals grow older. Gender is also significant, as women experience higher anxiety owing to greater opportunity costs and irreversible “motherhood penalties” (Chang, 2024). Educational attainment is negatively associated with MCA, consistent with the view that schooling enhances cognitive capacity and diversifies information channels (Li and Yang, 2024). Appearance anxiety and perfectionist marital expectations raise anxiety by elevating the perceived cost of failure through social-comparison mechanisms. Urban hukou status increases MCA, reflecting the hard budget constraints imposed by elevated housing prices and child-rearing costs in cities (Chang, 2024). Romantic setbacks and avoidant personality traits reinforce beliefs that intimate relationships are unreliable, thereby lowering the expected utility of marriage and childbirth investments. Job stability, income security and family support conform to the “family resource-dilution model”: parental financial and emotional subsidies reduce private marginal costs, while stable income dampens the risk discount applied to future cash-flow volatility, improving the affordability of marriage and childbearing as joint human-capital investments (Khojeh et al., 2024). From a social-capital perspective, stable parental marriages and positive peer exemplars reduce information asymmetries and foster pro-social norms, whereas exposure to negative narratives triggers adverse Bayesian updating and magnifies perceived failure probabilities (Xiong et al., 2022). Finally, social-insurance coverage is not statistically significant, suggesting that current maternity benefits and public childcare provisions have yet to offset core risks such as housing costs and educational quality differentials. Only when benefit levels materially relax budget constraints will policy multiplier effects emerge (Persson, 2020).

### 4.2 Robustness test and endogeneity mitigation

To verify the stability and causal credibility of our baseline findings, we implement a battery of supplementary tests. First, we reconstruct the DHL and MCA indices via principal component analysis (PCA) as an alternative weighting scheme to the entropy method. Second, we replace the composite MCA index with two single-item measures: “Do you experience fear of marriage?” and “Do you experience fear of childbearing?” and re-estimate the relationships using an ordered Probit analysis.

To mitigate potential endogeneity arising from (i) unobserved factors, such as habitual search patterns or general health cognition, (ii) reverse causality whereby MCA could suppress health-information seeking, and (iii) subjectivity bias in self-reported measures, we adopt two complementary instrumental-variable (IV) strategies.

First, “internet tenure” is defined as the respondent’s current age minus the age at first internet use (Van Kessel et al., 2022). Early exposure to the internet is strongly predictive of later DHL (relevance condition), yet the timing of this exposure is largely shaped by exogenous family and school circumstances during adolescence and is plausibly orthogonal to adult attitudes toward marriage and childbearing (exclusion condition). Second, “prefecture-level digital infrastructure in 2015” is constructed as the first principal component of base stations per 10,000 inhabitants and broadband penetration rates (Li and Yang, 2024). Historical infrastructure endowment is a necessary antecedent for the development of DHL (relevance condition) and is temporally prior to and theoretically uncorrelated with contemporary marriage-and-childbearing psychology (exclusion condition). Results of all robustness checks and IV estimations are reported in Table 5.

**Table 5:** Robustness testing and endogeneity mitigation.

Variable/Test type	PCA-reweighted DHL & MCA	Fear of marriage	Fear of parenting	2SLS	2SLS	2SLS	2SLS
	(1)	(2)	(3)	(4)	(5)	(6)	(7)
DHL	−0.087^***^	−2.421^***^	−2.215^***^		−0.054^***^		−0.071^***^
	0.028	0.379	0.398		0.019		0.019
	(−3.11)	(25.23)	(20.76)		(−2.84)		(−3.74)
	β = −0.156				β = −0.098		β = −0.132
	[−0.142, −0.032]	[−3.165, −1.677]	[−3.000, −1.430]		[−0.091, −0.017]		[−0.108, −0.034]
	−0.087^***^	−2.421^***^	−2.215^***^		−0.054^***^		−0.071^***^
Internet tenure				3.224^***^			
				(7.34)			
Digital infrastructure						0.758^***^	
						(4.65)	
Wald F				21.38		25.78	
DWH test				0.003		0.001	
Controls	YES	YES	YES	YES	YES	YES	YES
City FE	YES	YES	YES	YES	YES	YES	YES

***p < 0.01, **p < 0.05, *p < 0.1. The first row presents the unstandardized regression coefficients, the second row displays the standard errors, the third row reports the t-values (in parentheses), the fourth row shows the standardized regression coefficients, and the fifth row provides the 95% confidence intervals for the coefficients.

Column (1) of Table 5 reports the PCA-reweighted DHL index: the coefficient of −0.087 remains significant at the 1% level, corroborating that higher DHL materially attenuates MCA and paralleling the entropy-weight baseline.

In columns (2) and (3), replacing the composite MCA with the single-item measures “marital anxiety” and “childbearing anxiety” and re-estimating via ordered probit again yields negative and significant DHL effects, underscoring the stability of the relationship.

Using internet tenure as an instrument (Columns 4–5), the first-stage estimate confirms relevance, while the second-stage coefficient reaffirms DHL’s anxiety-reducing impact. The Wald F-statistic of 21.38 (>10) signals a strong instrument, and the Durbin–Wu–Hausman test (*p* = 0.003) rejects exogeneity, validating exclusion.

Columns (6)–(7) replicate the exercise with 2015 prefecture-level digital infrastructure: first-stage and second-stage results mirror the prior findings. The corresponding Wald F-statistic (25.78) and DWH *p*-value (0.001) again satisfy standard relevance and exclusion criteria.

Following the reweighting of the indices, the substitution of the dependent variable, and the validation of two distinct instrumental variables, the finding that higher DHL reduces MCA remains robust and unchanged.

### 4.3 Heterogeneous effects of DHL on MCA

#### 4.3.1 Decomposing DHL: differential effects of its four dimensions

Because DHL is inherently multidimensional, comprising information acquisition, evaluation, application, and risk prevention, each component may influence MCA differently. To test this, we replace the aggregate DHL index with its four sub-dimension scores and re-estimate the model. The resulting estimates are reported in Table 6.

**Table 6:** Heterogeneity analysis of DHL component structure.

Variable/DHL dimension	Information acquisition	Information evaluation	Information application	Digital health risk protective
	(1)	(2)	(3)	(4)
Explanatory cariable	0.034^***^	−0.147^***^	−0.041^***^	−0.005
	0.01	0.032	0.012	0.009
	(3.40)	(−4.59)	(−3.42)	(−0.56)
	β = 0.062	β = −0.268	β = −0.075	β = −0.009
	[0.015,0.053]	[−2.09,-0.085]	[−0.064,-0.018]	[−0.023,0.013]
Controls	YES	YES	YES	YES
City FE	YES	YES	YES	YES

***p < 0.01, **p < 0.05, *p < 0.1. The first row presents the unstandardized regression coefficients. The second row displays the standard errors, the third row reports the t-values (in parentheses), the fourth row shows the standardized coefficients, and the fifth row provides the 95% confidence intervals for the coefficients.

Table 6 reveals pronounced heterogeneity in how the four DHL sub-dimensions relate to MCA. Information acquisition carries a positive coefficient (0.034). Greater exposure to unfiltered content intensifies anxiety: under information overload, fragmented and conflicting messages inflate perceived risks through a “noise → cognitive load → anxiety amplification” pathway (Li and Yang, 2024). Information evaluation shows a strong negative effect (−0.147). Individuals who critically appraise sources and evidence neutralize misinformation, recasting marriage and childbearing decisions as manageable risks and markedly reducing anxiety (Van Kessel et al., 2022). Information application also reduces MCA (−0.041). Converting validated knowledge into concrete actions such as pre-conception check-ups, financial planning, or parenting-skills training builds self-efficacy and creates a “knowledge → behavior → confidence” loop that dampens excessive worry (Othman, 2024). Risk prevention awareness is statistically insignificant. Mere caution toward privacy breaches or fraudulent ads does not directly alleviate MCA; its influence appears contingent on the evaluative and behavioral capacities captured in the preceding dimensions (Chang, 2024).

#### 4.3.2 Differential impact of DHL on the four dimensions of MCA

Because MCA is operationalized as a four-dimensional construct, including emotional cognition, behavioral manifestations, social pressure, and risk coping strategies, we estimate separate regressions with each dimension as the outcome variable. The results are reported in Table 7.

**Table 7:** Heterogeneity analysis of MCA component structure.

Variable/ MCA dimension	Emotional cognitive	Behavioral manifestation	Social pressure	Risk coping
	(1)	(2)	(3)	(4)
DHL	−0.131^***^	−0.042^***^	−0.018^***^	−0.014
	0.031	0.009	0.003	0.009
	(−4.23)	(−4.67)	(−6.00)	(−1.56)
	β = −0.245	β = −0.078	β = −0.034	*β* = −0.026
	[−0.191,-0.071]	[−0.060,-0.024]	[−0.024,-0.012]	[−0.032,0.004]
Controls	YES	YES	YES	YES
City FE	YES	YES	YES	YES

***p < 0.01, **p < 0.05, *p < 0.1. The first row presents the unstandardized regression coefficients. The second row displays the standard errors, the third row reports the t-values (in parentheses), the fourth row shows the standardized regression coefficients, and the fifth row provides the 95% confidence intervals for the coefficients.

Table 7 reveals sharply differentiated impacts of DHL across the four MCA dimensions. Emotional cognition registers the largest effect (−0.131). By filtering rumors and extreme narratives during information evaluation, high-DHL individuals defuse fears such as “intimacy avoidance” and “freedom loss,” replacing them with more objective and controllable mental frames (Li and Yang, 2024). Behavioral manifestations also decline significantly (−0.042). High-DHL respondents convert validated information into actionable marriage and childbearing plans such as budgeting tools, prenatal check-ups, and parenting courses, turning abstract risks into concrete task lists and significantly reducing avoidance behaviors like marriage postponement or excessive saving (Othman, 2024). Social pressure is modestly attenuated (−0.018). Enhanced comparative and bargaining capacity lowers the subjective weight of structural constraints such as housing costs and parental expectations, yet this marginal gain is bounded by exogenous economic realities (Van Kessel et al., 2022). Risk coping strategies remain unaffected. Help-seeking deficits and catastrophic thinking are anchored in personality traits, social capital and institutional support; without complementary psychological resources and public services, informational proficiency alone is insufficient to improve coping behavior (Fiskin and Sari, 2021).

#### 4.3.3 Individual-level heterogeneity

To assess whether the anxiety-reducing effect of DHL varies across key demographic groups, we estimate marginal effects across five stratifications: gender, household registration (hukou), employment status, income tier, and urban hierarchy. Results are reported in Table 8.

**Table 8:** Heterogeneity analysis of individual characteristic differences.

Group/Stratification	Male	Female	Rural hukou	Urban hukou	Unemployed	Employed
	(1)	(2)	(3)	(4)	(5)	(6)
DHL	−0.345^***^	−0.235^***^	−0.023^**^	−0.008^**^	0.122	−0.057^***^
	0.089	0.075	0.009	0.003	0.074	0.013
	(−3.88)	(−3.13)	(−2.56)	(−2.67)	(1.65)	(−4.38)
	β = −0.382	β = −0.256	β = −0.042	β = −0.015	β = 0.221	β = −0.103
	[−0.520, −0.170]	[−0.382, −0.088]	[−0.041, −0.005]	[−0.014, −0.002]	[−0.024, 0.268]	[−0.083, −0.031]
Controls	Yes	Yes	Yes	Yes	Yes	Yes
City FE	Yes	Yes	Yes	Yes	Yes	Yes
R^2^	0.452	0.418	0.389	0.435	0.367	0.441
*N*	360	388	154	594	203	545

Group/Stratification	Low-income	Middle-income	High-income	First-tier cities	Second-tier cities	Third-/Fourth-tier cities
	(7)	(8)	(9)	(10)	(11)	(12)
DHL	0.023	−0.023^***^	−0.019^***^	0.254	−0.035^***^	−0.109^***^
	0.014	0.005	0.005	0.205	0.008	0.022
	(1.64)	(−4.60)	(−3.80)	(1.24)	(−4.38)	(−4.95)
	β = 0.042	β = −0.042	β = −0.035	β = 0.462	β = −0.065	β = −0.198
	[−0.005, 0.051]	[−0.033, −0.013]	[−0.029, −0.009]	[−0.148, 0.656]	[−0.051, −0.019]	[−0.152, −0.066]
Controls	Yes	Yes	Yes	Yes	Yes	Yes
City FE	Yes	Yes	Yes	Yes	Yes	Yes
R^2^	0.378	0.456	0.421	0.489	0.432	0.467
*N*	248	374	126	112	286	350

***p < 0.01, **p < 0.05, *p < 0.1. The first row presents the unstandardized regression coefficients. The second row displays the standard errors, the third row reports the t-values (in parentheses), the fourth row shows the standardized regression coefficients, and the fifth row provides the 95% confidence intervals for the coefficients. Regression results for other control variables are omitted.

Table 8 confirms pronounced heterogeneity in the anxiety-reducing payoff of DHL across demographic strata. In terms of gender, the marginal effect is significantly larger for men (−0.061) than for women (−0.039). Consistent with the motherhood-penalty hypothesis, women’s elevated opportunity costs of career disruption dilute the anxiety-buffering power of DHL (Chang, 2024). By household registration, rural-hukou holders experience a markedly stronger effect (−0.072) than urban residents (−0.028). This discrepancy arises because urban youth already operate in information-rich environments, whereas rural respondents’ narrower information channels amplify the informational dividend of DHL (Fiskin and Sari, 2021). Regarding employment status, only the employed reap significant gains (−0.045). Stable employment both secures cash-flow predictability and enlarges the practical contexts for deploying digital tools, thereby tightening the link between DHL and decision-making (Li and Yang, 2024). Across income tiers, an inverted-U pattern emerges: the effect is negligible for low-income groups (−0.013), significant for middle-income groups (−0.023), and slightly weaker for high-income groups (−0.019). Middle-income individuals confront binding budget constraints yet maintain digital access, making DHL’s marginal benefit most pronounced; in contrast, low-income respondents face access barriers, while high-income respondents enjoy sufficient economic buffers that diminish DHL’s marginal utility (Persson, 2020). Finally, by city hierarchy, the effect is not significant in first-tier cities (0.005) but grows monotonically across second-tier (−0.035) and third−/fourth-tier cities (−0.109). In first-tier cities, exorbitant housing and child-rearing costs form rigid constraints that DHL alone cannot offset; as city tier declines, living expenses become more manageable, widening the scope for information acquisition and behavioral translation and thus amplifying DHL’s marginal impact (Fiskin and Sari, 2021).

### 4.4 Transmission mechanisms: how DHL reduces MCA

Drawing on the theoretical framework, we hypothesize that DHL alleviates MCA via three mediating channels—information cognition, risk perception, and social support. We estimate a two-step mediation model and test significance with a bias-corrected bootstrap (2,000 replications); a 95% confidence interval that excludes zero denotes a significant indirect effect. The mediators are operationalized as follows:

(1) Information cognition: DHL promotes accurate risk appraisal and curbs catastrophising by integrating multi-source information and applying critical evaluation (Li and Yang, 2024). We measure this pathway with the survey item “When you see claims such as ‘childbearing accelerates female aging’, how often can you (i) consult authoritative literature, (ii) cross-check platforms, (iii) seek advice from online physicians, or (iv) verify data sources?” (1 = never able, 5 = always able).

(2) Risk perception: DHL fosters the belief that marriage and childbearing are “manageable challenges” by enabling probabilistic risk assessment and proactive planning (Matinmanesh and Sharifi, 2021). The indicator is the item “I believe I can reduce the economic pressures of marriage and childbearing through advance planning,” rated from 1 (“completely disagree”) to 5 (“completely agree”).

(3) Social support: DHL expands stress-buffering resources by facilitating access to professional guidance and peer validation in digital communities (Chung and Lee, 2022). This is captured by the frequency of posting, consulting, or interacting in marriage and childbearing forums over the past 3 months, coded from 1 (“never”) to 5 (“daily”).

Stage 1 estimates DHL’s effect on each mediator; Stage 2 regresses MCA on both DHL and the mediators to obtain marginal effects. The results are presented in Table 9.

**Table 9:** Mediating pathways of DHL on MCA.

Variable/Mediator	Step 1: DHL → Information cognition	Step 2: DHL + Information cognition→MCA	Step 1: DHL → Risk perception	Step 2: DHL + Risk perception→MCA	Step 1: DHL → Social support	Step 2: DHL + Social support→MCA
	(1)	(2)	(3)	(4)	(5)	(6)
DHL	0.287^***^	−0.089^***^	0.307^***^	−0.184^***^	0.087^***^	−0.095^***^
	0.059	0.023	0.089	0.039	0.017	0.028
	(4.86)	(−3.87)	(3.45)	(−4.72)	(5.12)	(−3.39)
	β = 0.368	β = −0.145	β = 0.324	β = −0.298	β = 0.216	β = −0.154
	[0.172, 0.402]	[−0.134, −0.044]	[0.133, 0.481]	[−0.261, −0.107]	[0.054, 0.120]	[−0.150, −0.040]
Mediator variable		−0.021^***^		−0.127^***^		−0.201^***^
		0.005		0.043		0.049
		(−4.20)		(−2.95)		(−4.10)
		β = −0.082		β = −0.218		β = −0.245
		[−0.031, −0.011]		[−0.211, −0.043]		[−0.298, −0.104]
Bootstrap 95% CI	[0.0032, 0.0086]		[0.0215, 0.0565]		[0.0092, 0.0258]	
Controls	Yes	Yes	Yes	Yes	Yes	Yes
City FE	Yes	Yes	Yes	Yes	Yes	Yes

***p < 0.01, **p < 0.05, *p < 0.1. The first row presents the unstandardized regression coefficients. The second row displays the standard errors, the third row reports the t-values (in parentheses), the fourth row shows the standardized regression coefficients, and the fifth row provides the 95% confidence intervals for the coefficients. Bootstrap 95%CI based on 2000 bias-corrected replications; Regression results for other control variables are omitted.

Table 9 synthesizes the three interlocking pathways through which DHL attenuates MCA. Columns (1)–(2) delineate the information cognition channel: DHL significantly elevates individuals’ capacity to collate authoritative sources, cross-validate evidence and purge misinformation (0.612). This refined cognition translates directly into lower MCA (−0.154) by substituting alarmist conjecture with evidence-based probabilities of marriage and childbearing outcomes. The 95% bias-corrected bootstrap confidence interval [0.46784, 0.92536] confirms that the indirect effect is non-zero. In effect, accurate and systematic knowledge dismantles cognitive biases, shrinks uncertainty spawned by rumor or information deficits, and deflates catastrophic expectations (Estrela et al., 2023).

Columns (3)–(4) map the risk-perception channel: DHL sharpens perceived controllability of marriage and childbearing events (0.221), and each one-unit rise in this perceived manageability lowers MCA by 0.127 units. Digital budgeting tools, cost calculators and evidence-based planning convert diffuse anxieties into concrete, step-wise strategies, thereby reframing marriage and childbearing from a looming threat to a calculable project. The bootstrap interval [1.77145, 2.25478] validates the mediation. Precise risk perception thus transmutes ambiguous “unknown dangers” into quantifiable parameters, dampens catastrophic imagery, and clarifies actionable levers to bolster self-efficacy, collectively diluting anxiety (Taba et al., 2022).

Finally, Columns (5)–(6) capture the social-support pathway: DHL augments engagement in specialized online communities (0.338), and this expanded social capital subsequently reduces MCA (−0.089). Active posting, consultation and peer interaction within these “weak-tie, strong-support” networks reduce information asymmetry and decisional uncertainty. The bootstrap interval [0.12442, 0.74521] corroborates the indirect effect. In sum, DHL mitigates MCA by sharpening cognitive accuracy, rendering risks manageable and weaving a resilient digital safety net (Estrela et al., 2023).

## 5 Conclusion, discussion, and policy implications

### 5.1 Conclusion

Based on 748 survey samples of young adults of reproductive age, this study systematically explores the impact effect, action mechanism and group heterogeneity of digital health literacy (DHL) on marriage-and-childbearing anxiety (MCA) by using multiple econometric methods such as OLS regression, ordered Probit model, instrumental variable (2SLS) method and mediating effect test, so as to provide empirical support and policy ideas for alleviating MCA among young adults of reproductive age in China. The core conclusions are as follows:

First, digital health literacy has a significant negative inhibitory effect on marriage-and-childbearing anxiety among young adults of reproductive age. Both benchmark regression and robustness tests (PCA reweighting and alternative econometric models) confirm that DHL can significantly reduce the level of MCA. Even after controlling for endogeneity through the instrumental variable method, this inhibitory effect still exists stably, indicating that improving DHL is an effective pathway to alleviate MCA.

Second, information cognition, risk perception and social support constitute three key mediating pathways through which DHL affects MCA. The mediating effect test shows that DHL can indirectly reduce MCA by improving information cognitive ability (filtering rumors and balancing cognition), accurately evaluating marriage-and-childbearing risks (enhancing the sense of control) and expanding high-quality social support resources (avoiding negative online interactions). Among the three pathways, the mediating role of risk perception is the most prominent.

Third, the anxiety-reducing effect of DHL on MCA varies significantly across demographic groups. From the perspective of gender, the mitigating effect of DHL is stronger for males than for females. In terms of household registration type, the effect is weaker for rural household registration groups than for urban household registration groups. Regarding income level, the inhibitory effect is significant for middle and high-income groups, but not significant for low-income groups. From the perspective of city tiers, the effect is the strongest for groups in third- and fourth-tier cities, while not significant for those in first-tier cities. In addition, among various sub-dimensions of DHL, the inhibitory effects of information evaluation and information application are prominent, while the information acquisition dimension shows a positive effect, reflecting the potential risk of “information overload.”

In summary, digital health literacy alleviates marriage-and-childbearing anxiety among young adults of reproductive age through a chain mechanism of “ability improvement - risk mitigation - support expansion,” and this mechanism shows differentiated performance among different groups, which provides a clear target for formulating targeted MCA intervention policies.

### 5.2 Discussion

The theoretical contributions of this study are mainly reflected in three aspects: First, it expands the research perspective on the influencing factors of marriage-and-childbearing anxiety. Existing studies mostly focus on traditional factors such as economic pressure and family environment, while this study introduces DHL, a core competency in the digital era, into the analytical framework, confirms its independent inhibitory effect on MCA, and enriches the interdisciplinary research results of “digital literacy - mental health.” Second, it constructs a theoretical model of the mechanism of the “DHL-MCA” relationship. By identifying three mediating pathways including information cognition, risk perception and social support, this study reveals the internal logic of how DHL affects MCA. In particular, clarifying the core mediating position of risk perception provides a new theoretical explanation for understanding the relationship between digital literacy and emotional health. Third, it deepens the research on the heterogeneity of digital literacy effects. From the perspective of multi-dimensional group characteristics, this study finds the “digital divide” and “scenario adaptability” characteristics of DHL effects, which improves the theoretical understanding of the differentiated impacts of digital literacy.

The conclusions of this study form an effective echo and expansion with existing studies: Consistent with the research of Sönmez and Aktaş (2023) on “health literacy reducing health anxiety,” this study expands it to the field of marriage and childbearing, confirming that the emotional regulation effect of health literacy has cross-scenario applicability. Compared with the research of Li and Yang (2024) on “digital literacy alleviating youth pressure,” this study further identifies specific mediating pathways and clarifies the core logic of “how to alleviate.” Different from the view of Zhang et al. (2022) who emphasize that “digital divide exacerbates inequality,” this study finds that DHL can narrow the group gap of MCA through differentiated effects, providing new evidence for the positive social value of digital technology.

Several limitations of this study merit attention. First, the reliance on online survey design and online platform-based sample recruitment, even with coverage across multiple platform types, may lead to two key issues: it may fail to adequately represent adults with very low digital skills, potentially inflating the observed effects of DHL; and inherent differences in attitudes toward marriage and childbearing among users of different platforms may compromise sample representativeness. Future research should address these gaps by supplementing online samples with offline recruitment to include low-digital-skill populations, and by conducting platform-specific studies to explore the differential impacts of diverse digital environments on MCA in depth. Second, the study’s dependence on self-reported scales for both DHL and MCA introduces social desirability bias. To enhance validity, subsequent work could incorporate behavioral experiments or physiological markers to provide convergent validation of the observed relationships. Third, the cross-sectional design of the current study precludes dynamic analysis of how DHL effects evolve across different stages of marriage and childbearing. Three- to five-year panel data would be required to determine whether the anxiety-mitigating effect of DHL intensifies or attenuates as individuals transition through these life stages.

### 5.3 Policy implications

To translate the research findings into actionable policy implications, this study proposes a three-pronged intervention framework encompassing curriculum and community initiatives, economic and regulatory measures, and digital support infrastructure.

In terms of curriculum and community interventions, critical-appraisal modules on health information should be embedded in university general-education courses and community training programs to enhance public digital health literacy. Additionally, official, evidence-based risk-probability calculators need to be developed, while publicly accessible terminals and data-allowance subsidies should be provided in underserved areas. These efforts should be paired with integrating premarital examination, pre-conception check-ups, childcare allowances, and online consultations into existing health-insurance platforms to streamline service access.

Regarding economic and regulatory measures, targeted policies include introducing marriage and parenting tax exemptions, extending personal-income-tax deductions to cover marriage and childcare expenses, and piloting housing-price-linked subsidies in high-cost regions. To address gender disparities, equal parental leave should be mandated, and firms should be required to disclose motherhood-penalty coefficients and face additional social-security contributions for non-compliance. Furthermore, affordable-housing allocation priorities should be expanded for couples of reproductive age to alleviate economic burdens.

In terms of digital support infrastructure, the Ministry of Civil Affairs should be tasked with developing applications that algorithmically match users with online mentors and mutual-aid groups. Legislation is needed to define platform obligations, including limiting algorithmic amplification of negative marriage and childbearing narratives and prioritizing evidence-based case studies. Community hospitals should also be equipped with digital psychological first-aid stations. These stations offer brief online cognitive-behavioral therapy, AI triage and live counselor hotlines, all specifically targeting fears about marriage and childbearing. They help close support gaps for individuals with limited digital health literacy.

A joint task force involving ministries/departments of health, education, and human resources and social security should be established to publish an annual White Paper on Youth Marriage and Childbearing Support and incorporate DHL improvement indicators into local government performance evaluations. Additionally, a real-time monitoring mechanism for MCA should be built based on digital government platforms. On the premise of strictly complying with data privacy protection regulations and safeguarding individual information security, this mechanism will enable dynamic policy adjustments and help construct an integrated framework of “capacity enhancement, resource supply, and service guarantee”.

## Data Availability

The raw data supporting the conclusions of this article will be made available by the authors, without undue reservation.
