# Supplementary material for: The impact of digital health literacy on marriage-and-childbearing anxiety among Chinese youth of reproductive age
Source: Front Psychol. 2025 Nov 27;16:1676542. doi: 10.3389/fpsyg.2025.1676542 (PMC12695770; doi:10.3389/fpsyg.2025.1676542)
Supplement: Supplementary file 1 [file Table_1.docx]

Appendix Table 1 Reliability and Validity Test Results of DHL and MCA Scales

| Construct | Dimension | No. of Items | Cronbach’s α | Composite Reliability (CR) | Average Variance Extracted (AVE) | Factor Loading Range |
| --- | --- | --- | --- | --- | --- | --- |
| Digital  Health  Literacy  (DHL) | Information Acquisition | 4 | 0.826 | 0.843 | 0.512 | 0.632–0.785 |
|  | Information Evaluation | 4 | 0.879 | 0.901 | 0.634 | 0.715–0.856 |
|  | Information Application | 4 | 0.857 | 0.882 | 0.589 | 0.683–0.812 |
|  | Risk Prevention | 4 | 0.815 | 0.823 | 0.508 | 0.618–0.764 |
|  | Overall Scale | 16 | 0.876 | 0.895 | 0.567 | — |
| Marriage  And  Childbearing Anxiety (MCA) | Emotional-Cognitive | 9 | 0.892 | 0.913 | 0.578 | 0.692–0.834 |
|  | Behavioral Manifestation | 9 | 0.885 | 0.906 | 0.562 | 0.675–0.821 |
|  | Social Pressure | 9 | 0.864 | 0.887 | 0.537 | 0.641–0.798 |
|  | Risk-Coping | 9 | 0.878 | 0.902 | 0.559 | 0.663–0.815 |
|  | Overall Scale | 36 | 0.902 | 0.924 | 0.658 | — |

Notes: 1. Cronbach’s α > 0.8 indicates good reliability; CR > 0.8 and AVE > 0.5 indicate good convergent validity. 2. Discriminant validity test: The square root of AVE for each dimension is greater than the correlation coefficient between dimensions.

Appendix Table 2 Common Method Bias Test Results (Harman’s Single-Factor Test)

| Factor Number | Eigenvalue | Variance Explained (%) | Cumulative Variance Explained (%) |
| --- | --- | --- | --- |
| 1 | 22.89 | 32.7 | 32.7 |
| 2 | 9.442 | 13.489 | 46.189 |
| 3 | 6.252 | 8.931 | 55.12 |
| 4 | 4.945 | 7.064 | 62.184 |
| 5 | 3.863 | 5.519 | 67.703 |
| 6 | 3.352 | 4.789 | 72.492 |
| 7 | 2.835 | 4.05 | 76.542 |
| 8 | 2.495 | 3.564 | 80.106 |
| 9 | 2.325 | 3.322 | 83.428 |
| 10 | 2.148 | 3.069 | 86.497 |
| 11 | 2.058 | 2.94 | 89.437 |
| 12 | 2.007 | 2.867 | 92.304 |

Notes: 1. The test covers all 70 measurement items in the questionnaire, including 16 items of the Digital Health Literacy (DHL) scale (4 dimensions × 4 items each), 36 items of the Marriage-and-Childbearing Anxiety (MCA) scale (4 dimensions × 9 items each), and 18 key items of control variables. 2. Exploratory Factor Analysis (EFA) was conducted without rotation, and factors with eigenvalues greater than 1 were extracted. 3. The variance explained by the first common factor is 32.7%, which is lower than the critical threshold of 40%, indicating that there is no severe common method bias in this study.
